# Supplementary material for: Direct Evidence on Effect of Oxygen Dissolution on Thermal and Electrical Conductivity of AlN Ceramics Using Al Solid-State NMR Analysis
Source: Materials (Basel). 2022 Nov 16;15(22):8125. doi: 10.3390/ma15228125 (PMC9695506; doi:10.3390/ma15228125)
Supplement: Supplementary file 1 [file materials-15-08125-s001.zip › materials-2004869-supplementary.pdf]

materials

Supplementary Materials

Direct Evidence on Effect of Oxygen Dissolution on Thermal and Electrical Conductivity of AlN Ceramics using Al Solid-State NMR Analysis

Jaegyeom Kim [1,†], Jong-Young Kim [1,†], Heewon Ahn [1], Mu Hyeok Jeong [1,2], Eunsil Lee [1,2], Keonhee Cho [1], Sung-Min Lee [1], Wooyoung Shim [2] and Jae-Hwan Pee [1,*]

[1] Icheon Branch, Korea Institute of Ceramic Engineering and Technology(KICET), 3321, Gyeongchung Rd., Sindun-Myeon, Icheon-si, Gyeonggi-do 467-843, Republic of Korea
[2] Department of Materials Sciences & Engineering, Multi-scale Materials Laboratory, Yonsei University, 50, Yonsei-ro, Seodaemun-gu, Seoul 37022, Republic of Korea
* Correspondence: pee@kicet.re.kr; Tel.: +82-31-645-1423
† The authors equally contributed as the first author.

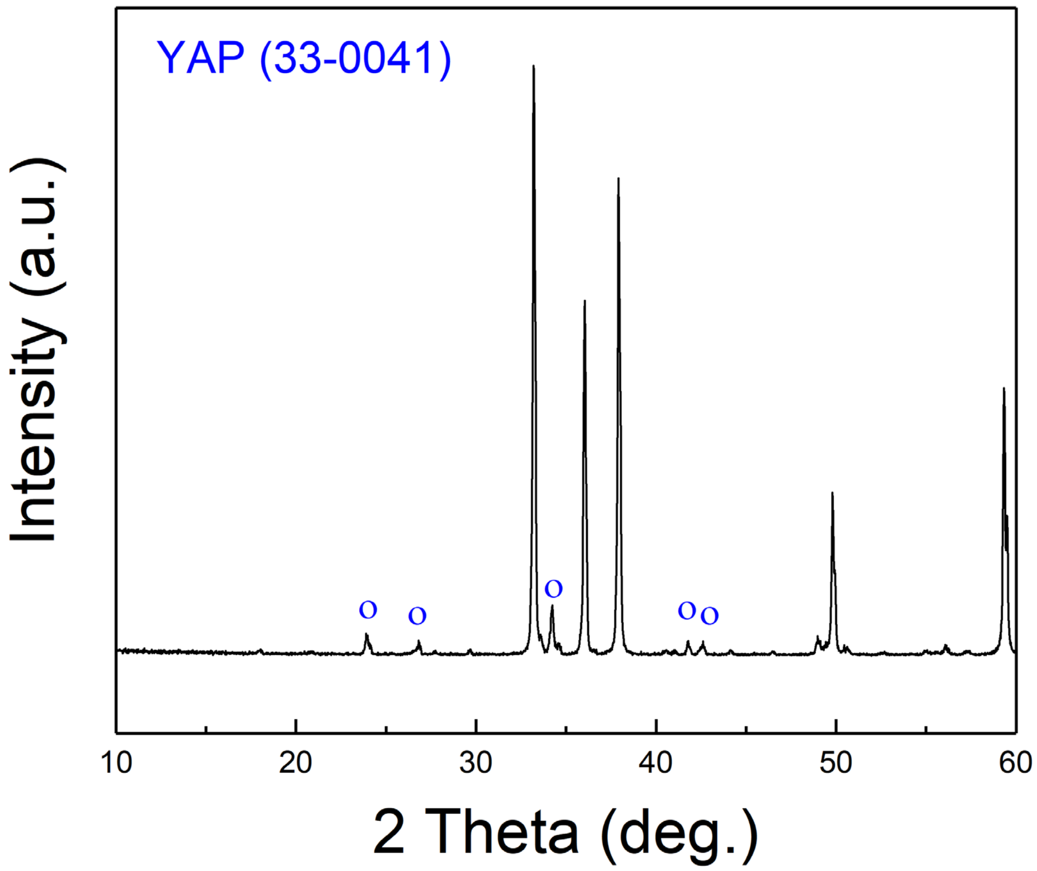

Figure S1. XRD pattern of sintered commercial AlN.

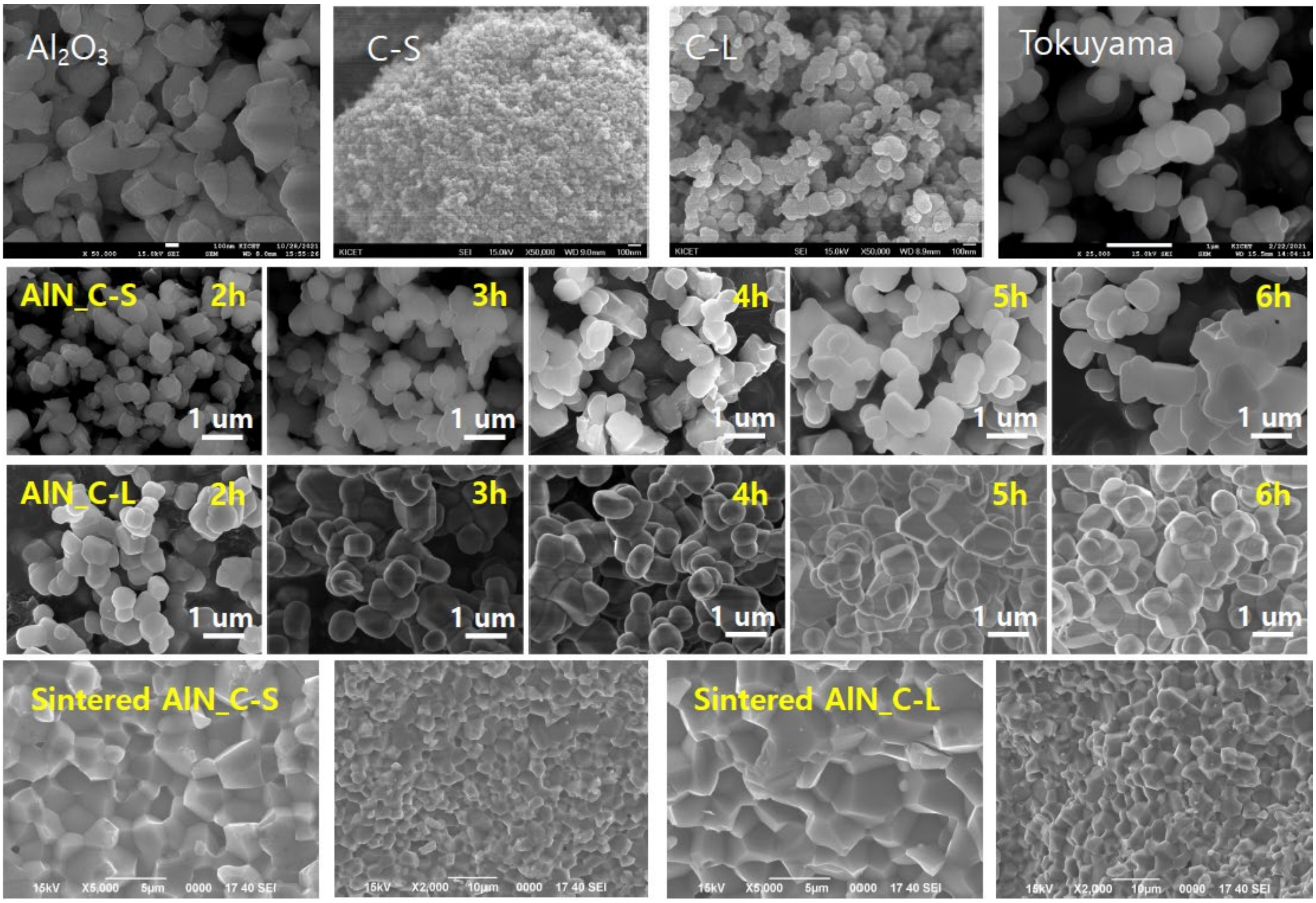

Figure S2. SEM images for Al2O3, carbon black, AlN raw materials, and sintered AlN.

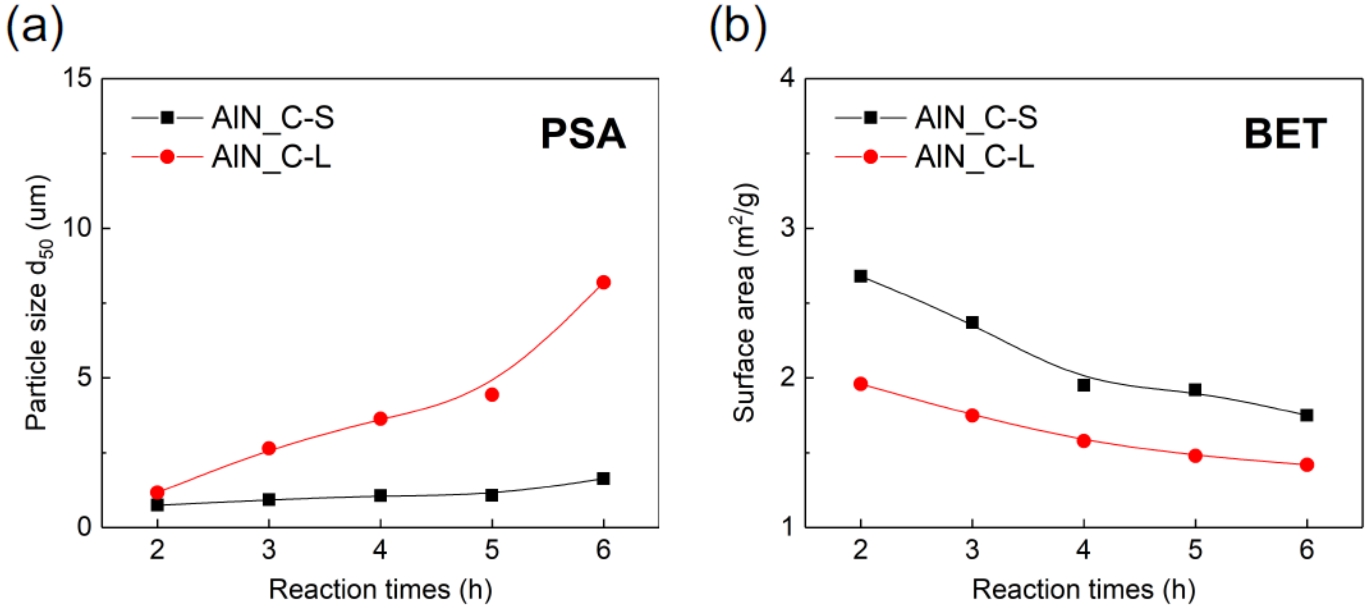

Figure S3. Particle size and BET data of AlN_C-S/C-L.
